# Draft Genome Sequence of *Megasphaera* sp. Strain DJF_B143, an Isolate from Pig Hindgut Unable to Produce Skatole

**DOI:** 10.1128/genomeA.00007-16

**Published:** 2016-02-25

**Authors:** Xiaoqiong Li, Ian P. G. Marshall, Lars Schreiber, Ole Højberg, Nuria Canibe, Bent Borg Jensen

**Affiliations:** a Department of Animal Science, Aarhus University, Tjele, Denmark; b Center for Geomicrobiology, Department of Bioscience, Aarhus University, Aarhus, Denmark

## Abstract

The butyrate-producing *Megasphaera* spp. predominate in the pig hindgut and may play important roles in gut health. Moreover, one *Megasphaera* isolate has been reported to produce the boar taint compound, skatole. Here, we provide a 2.58-Mbp draft genome of a pig hindgut isolate, *Megasphaera* sp. DJF_B143, unable to produce skatole.

## GENOME ANNOUNCEMENT

Bacteria of the genus *Megasphaera* are normal inhabitants of the gastrointestinal tract of mammals (1–4) and can constitute a considerable proportion of the microbial community in intestinal contents of pigs (5, 6). Due to its capability to convert lactate to butyrate, the type species *M. elsdenii* is a probiotic candidate for animals (7). Additionally, *Megasphaera* sp. TrE9262, isolated from sheep, has been reported to produce skatole (3-methylindole) (8), a main contributing compound of boar taint (9). We have isolated strain DJF_B143 belonging to the genus *Megasphaera* from pig hindgut content. According to 16S rRNA gene sequence analysis, strain DJF_B143 (EU728714) is only distantly related to the described *Megasphaera* spp., like *M. indica* (HM990964, 94.0% identity) and *M. micronuciformis* (GU470904, 93.9% identity), and its closest phylogenetic relative is *Megasphaera* sp. TrE9262 (DQ278866, 99.9% identity). We confirmed the production of butyrate, but observed no skatole production by DJF_B143 either in modified peptone-yeast-glucose or in colon fluid-glucose-cellobiose-agar (CGCA) media. We sequenced strain DJF_B143 to explore the genomic basis for these metabolic observations.

*Megasphaera*. sp. DJF_B143 was grown anaerobically in CGCA at 37°C. Genomic DNA was isolated using a Maxwell 16S DNA purification kit (Promega, USA), and automated DNA purification was performed on a Maxwell 16 Instrument (Promega). A sequencing library was prepared using the Nextera XT kit (Illumina, USA). Genome sequencing was performed using the Illumina MiSeq platform with a paired-end 300-bp MiSeq reagent kit version 3. The resulting sequence reads (ca. 1.7 million read pairs; ≈1 Gbp) were inspected for data quality using FastQC version 0.10.1 (http://www.bioinformatics.babraham.ac.uk/projects/fastqc). Reads were trimmed using Trimmomatic version 0.32 (10) with the following parameters: CROP:250, HEADCROP:20, SLIDINGWINDOW:4:20, and ILLUMINACLIP:adapters.fasta:2:40:15 MINLEN:100. Trimmed Reads were assembled using SPAdes version 3.6.1 (11) with the following parameters: ‑k 21,33,55,77,99,127 –careful. Contigs most likely originating from contamination were removed using MetaWatt version 3.5 (12). The decontaminated reads mapping to the retained *Megasphaera* contigs (ca. 1.38 million read pairs; ≈0.53 Gbp) were extracted using BBMap version 34.94 (http://sourceforge.net/projects/bbmap/files) with the “minid = 0.98” option. The extracted reads were assembled using SPAdes as before, generating 32 contigs (>200 bp) with ca. 204× coverage. The draft genome sequence of DJF_B143 has a total length of 2,581,251 bp, an average G+C content of 49.5%, and an *N_50_* length of 161,321 bp. CheckM version 1.0.3 (13) estimated the genome to be 99.9% complete when using the gene marker set for the genus *Megasphaera*.

Prokka version 1.1 (14) identified 2,297 protein-coding sequences, 9 rRNAs (including 7 5S, 1 16S, and 1 23S) and 56 tRNAs. The absence of an aliphatic amidase (EC 3.5.1.4) gene in DJF_B143 suggests that this strain lacks the metabolic pathway from tryptophan to skatole via indole-3-acetate (15). Strain DJF_B143 possesses the genomic potential to produce butyrate from acetate via butyryl coenzyme A (CoA):acetate-CoA transferase (But) (EC 2.8.3.8).

### Nucleotide sequence accession numbers.

The *Megasphaera* sp. DJF_B143 genome sequence has been deposited at DDBJ/EMBL/GenBank under the accession number LODR00000000. The version described in this paper is the first version, LODR01000000.
